# Clinical Cases

**Published:** 1890-06

**Authors:** Ella M. Tuttle

**Affiliations:** New Berlin, New York


### CLINICAL CASES.

Ella M. Tuttle, M. D., New Berlin, New York.

Some Effects of Quinine.—Was called to see Miss ——, whom I found suffering from eczema of the hands. She stated that she had been afflicted with the same disease before when living in a distant State, and as at that time she suffered from it for about five weeks, she was expecting a long siege. On inquiry I learned that at that time she had been first under an allopathic physician, who had given her quantities of Quinine, etc., but growing steadily worse she had finally called upon a homœopathic physician, under whose treatment she had recovered. Before I saw her at this time she had been taking Quinine for a cold, but when the eczema appeared she sent for me. The skin of her hands was reddened, and somewhat excoriated, itched violently, and burned after scratching. The joints of the fingers were lame and stiff and would pain her severely if kept long in one position, but were momentarily relieved by moving. Gave her Rhus-tox., under which, to her surprise, she recovered in a few days.

Some time after she had a cold, and again began taking Quinine. In two hours after the first dose her hands began to burn and itch in the same way, but this time she recognized that it was the effect of the Quinine, and immediately stopped its use. This time the itching and burning only lasted about twenty-four hours. When will people learn that indefinite dosing with Quinine is at the bottom of a large per cent. of the ills that afflict us poor mortals?

Arsenicum in the Vomiting of Pregnancy.—Mrs. F——, one month pregnant, was taken with a distressing attack of vomiting. Morphine was given her, both by the mouth and injected into her arm, but with the effect of increasing the vomiting. I was called after two days and nights of suffering. Found her very pale and weak, hardly able to raise her head from the pillow. Would vomit about once in twenty minutes, the vomited matter consisting of white frothy mucus. Said she felt very nervous and feared she was going to die. Began to have labor pains, and the physician she had before thought she would lose her baby.

I put ten drops of Ars.$^{3}$ in half a glass of water, and gave her a spoonful every half-hour. The first spoonful was promptly ejected, but the next was retained and the vomiting effectually checked. In the course of two hours she was able to take some beef-tea, and the fourth day after she was in the kitchen superintending her hired help. She went to term, at which time I had the pleasure of delivering her of a healthy eight-pound girl.

